# Examining the relationship between cardiometabolic risk factors and telomere length in women: a systematic review

**DOI:** 10.1093/geroni/igaf091

**Published:** 2025-08-25

**Authors:** Jeni Page, Catherine Stephens, Melissa A Richard, Elizabeth Lyons, Elizabeth Baumler, M Terese Verklan, Elizabeth Lorenzo

**Affiliations:** School of Nursing, University of Texas Medical Branch, Galveston, Texas, United States; School of Nursing, University of Texas Medical Branch, Galveston, Texas, United States; Department of Pediatrics, Baylor College of Medicine, Houston, Texas, United States; School of Health Professions, University of Texas Medical Branch, Galveston, Texas, United States; McGovern Medical School, University of Texas Health Science Center at Houston, Houston, Texas, United States; School of Nursing, University of Texas Medical Branch, Galveston, Texas, United States; School of Nursing, University of Texas Medical Branch, Galveston, Texas, United States

**Keywords:** Obesity, Dyslipidemia, Hypertension, Insulin resistance

## Abstract

**Background and Objectives:**

Cardiometabolic syndrome (CMS) poses a significant public health challenge due to its rising prevalence in aging and significant healthcare costs. Recent studies have suggested telomere length (TL), a marker of cellular aging, may be impacted by CMS among women, but comprehensive evidence remains limited. This study aimed to examine the association between CMS risk factors (increased waist circumference [WC], elevated blood pressure, impaired fasting blood glucose, elevated triglycerides, decreased high-density lipoproteins) and TL in women, with consideration of age and race or ethnicity.

**Research Design and Methods:**

A systematic review was conducted following PRISMA guidelines, with searches across five databases. Thirteen relevant studies published between 2007 and 2022 were included. A narrative synthesis was performed to evaluate associations between CMS risk factors and TL.

**Results:**

Findings revealed individual CMS risk factors did not demonstrate relationships with TL; however, a link was identified between collective CMS risk factors and decreased TL. The influence of CMS on TL varied by mean sample age, where increased WC was associated with decreased TL for middle adulthood women. Findings based on race or ethnicity were inconclusive due to limited analyses, but examination by continent revealed a relationship between increased WC and decreased TL in Asia and North America.

**Discussion and Implications:**

There was high heterogeneity among diagnostic criteria for CMS risk factors across studies, potentially limiting findings. This review highlights the need for further research to clarify the complex associations between CMS and TL in women throughout the lifespan. Future large cohort studies using standardized CMS diagnostic criteria should examine variations by age and race or ethnicity to enhance understanding of these relationships.

Translational Significance Cardiometabolic syndrome (CMS) accelerates biological aging and increases chronic disease risk in women, yet its role across the life course remains unclear. This systematic review synthesizes evidence showing that CMS risk factors, particularly increased waist circumference during middle adulthood, are associated with shorter telomere length (TL), a marker of cellular aging. These findings are innovative in identifying a critical period linking CMS and cellular aging, bridging life course epidemiology with molecular biomarkers. They can be translated into clinical and public health strategies, such as targeted screening and prevention, to promote healthy aging in women.

## Background and objectives

Cardiometabolic syndrome (CMS) represents a cluster of five risk factors, including increased waist circumference (WC), elevated blood pressure (BP), impaired fasting blood glucose (FBG), increased triglycerides (TG), and decreased high-density lipoproteins (HDL) (Grundy et al., 2004; NCEP Expert Panel, 2001). Individuals with CMS face an elevated risk of developing cardiovascular diseases, such as heart attack, stroke, and peripheral artery disease (National Heart, Lung, and Blood Institute, 2022). Moreover, this population faces a heightened risk of adverse health outcomes and experiences significantly increased annual healthcare costs compared to individuals without CMS (McQueen et al., 2016; NCEP Expert Panel, 2001).

The diagnosis of CMS involves cutoff values that vary based on multiple existing diagnostic criteria, introducing complexity into efforts to calculate the global burden of the condition accurately. Despite these challenges, data reveal a substantial increase in CMS prevalence in recent decades, particularly affecting women, with approximately 32 million women diagnosed with CMS in the United States (Cheng et al., 2022a). The incidence escalates with age, with nearly 50% of women 60 years or older affected (Hirode & Wong, 2020). There are also notable disparities in CMS prevalence by race or ethnicity, with Hispanic (Hirode & Wong, 2020) and non-Hispanic Black women (American Heart Association, 2023) exhibiting higher rates of CMS compared to non-Hispanic White women.

Telomeres are repetitive DNA sequences at the ends of chromosomes that protect them from degradation and prevent them from fusing with neighboring chromosomes (Blackburn, 1991). These structures play a pivotal role in cellular aging and lifespan, gradually shortening with advancing age (Shammas, 2011). The shortening of telomere length (TL) is intricately linked with various chronic diseases, including CMS (Fitzpatrick et al., 2007; Loh et al., 2021). This connection is attributed to oxidative stress and chronic inflammation, two causal mechanisms associated with CMS development (Sverdlov et al., 2016; von Zglinicki, 2002). Thus, examination of TL poses a potential disease-specific molecular marker that may be utilized when investigating CMS prevalence (Yeh & Wang, 2016).

The relationship between CMS and TL has been addressed in previous studies, which have linked shorter TL to increased abdominal obesity (Garcia-Calzon et al., 2014; Lee et al., 2011) and elevated BP (Demissie et al., 2006; Huang et al., 2020). Additionally, research has established a connection between shortened TL and the progression of insulin resistance (Gardner et al., 2005), as well as increased risk of glycemic progression in individuals already diagnosed with type 2 diabetes (Cheng et al., 2022b). Increased risk of TL shortening has been reported in individuals with higher concentrations of TG (Karimi et al., 2019), and significant associations have been identified between decreased HDL and decreased TL as well (Liu et al., 2023). However, there is evidence that some CMS risk factors may have longitudinal effects that help mitigate aspects of cellular aging when managed effectively. For instance, controlling blood pressure and maintaining healthy lipid levels may reduce the rate of telomere shortening, thereby potentially decelerating cellular aging (Liu et al., 2023). These findings underscore the need for further investigation to fully understand the biological pathways involved and the long-term implications for aging-related health outcomes.

There are some notable sex and race- or ethnic-specific variations in CMS risk factor prevalence (Lear & Gasevic, 2019) and TL (Needham et al., 2019; Roux et al., 2009). Research has shown that women experience a steeper age-related increase in CMS prevalence compared to men, along with a higher prognostic significance for cardiovascular disease and mortality (Meloni et al., 2023). Additionally, CMS often presents differently between the sexes, with women more likely to exhibit increased WC than men (De Jong et al., 2020; Prasad et al., 2020). This is further supported by sex-specific cutoff points highlighting variations in the diagnostic criteria for two of the five risk factors (Huang, 2009). The hormonal changes accompanying perimenopause and menopause, particularly the reduction in estrogen, have been implicated in increased visceral fat accumulation, impaired insulin sensitivity, and heightened inflammation, all of which contribute to elevated CMS risk (Alemany, 2021; Meloni et al., 2023). Beyond its metabolic effects, estrogen has been shown to preserve TL by reducing oxidative stress and enhancing telomerase activity (Kresovich et al., 2018; Viña et al., 2005), suggesting a biological link between hormonal changes, CMS, and cellular aging. Together with genetic predisposition (Tramunt et al., 2020) and lifestyle factors (Mauvais-Jarvis et al., 2020), these sex-specific biological mechanisms may underscore the importance of incorporating hormonal and life-stage considerations in research on CMS and TL in women.

In the examination of race and ethnicity, Lear and Gasevic (2019) observed that the majority of data on the management and prevention of CMS and its risk factors are predominantly based on studies involving European-derived populations. This is concerning because diagnostic thresholds, particularly for measures of WC, have been shown to vary significantly across different racial and ethnic groups. With TL, non-Hispanic Black individuals often are reported to have longer TL than non-Hispanic White individuals (Needham et al., 2019; Roux et al., 2009). Moreover, much of the published literature on CMS and TL has been limited to only males (Demissie et al., 2006; Karimi et al., 2019), includes mostly non-Hispanic White populations (Demissie et al., 2006; Lee et al., 2011; Révész et al., 2014), is limited to a specific regional population (Garcia-Calzon et al., 2014; Liu et al., 2023), and/or does not include separate subanalyses by sex (Lee et al., 2011). Furthermore, prior systematic reviews have predominantly focused on one of the five CMS risk factors and TL (Mundstock et al., 2015; Zadeh et al., 2023), with none examining all five risk factors or CMS as the outcome. Additionally, no review to date has focused exclusively on women, despite their higher prevalence of CMS and distinct biological and sociodemographic risk profiles.

The purpose of this review is to address the significant gap in understanding the sex-specific associations between CMS as a clinical condition, its individual CMS risk factors, and TL in women. By examining both CMS and its component risk factors, specifically in women, we aim to provide a comprehensive understanding of how these elements, influenced by biological, hormonal, and sociodemographic factors, are associated with TL, particularly in the context of age and race or ethnicity.

## Research design and methods

This systematic review is registered with the International Prospective Register of Systematic Reviews (https://www.crd.york.ac.uk/prospero/; CRD42023407309) and adheres to the guidelines of Preferred Reporting Items of Systematic Reviews and Meta-Analyses (PRISMA) (Moher et al., 2009).

### Data sources and searches

An extensive literature search was conducted from March to April 2023 and updated in January 2025 using five databases: PubMed, OVID, Scopus, CINAHL, and Web of Science. No limitations were set on publication dates to provide a more comprehensive overview of past research. The search string was uniformly entered into each database for consistency: (metabolic syndrome OR cardiometabolic syndrome OR cardiometabolic risk factors OR abdominal obesity OR increased waist circumference OR atherogenic dyslipidemia OR decreased high-density lipoprotein OR elevated triglycerides OR hypertension OR prediabetes OR hyperglycemia OR insulin resistance OR elevated fasting blood glucose) AND (women OR female) AND telomere. Inclusion criteria required studies to be published in English within peer-reviewed journals, excluding unpublished studies, conference abstracts, and review articles. Two reviewers (JP, CS) autonomously conducted searches across the databases. Titles were collated and entered into Covidence Systematic Review software (Veritas Health Innovation, 2023). Duplicate entries were eliminated upon import and verified by the reviewers. Both reviewers (JP, CS) independently performed a full-text review for potential inclusion in the review. Additionally, reference lists of the included articles and other relevant systematic reviews were manually searched to identify supplementary studies for inclusion.

### Study screening and selection

The screening procedure entailed an assessment of study titles and abstracts by both reviewers (JP, CS) to determine inclusion following the PICO framework (population—women, intervention—CMS and/or CMS risk factors, comparator—any or none, outcome—telomere length). Inclusion criteria were (1) examination of outcomes in adult females aged 18 years and older; (2) utilization of quantitative research designs, including descriptive, correlational, causal-comparative/quasi-experimental, and experimental designs; (3) evaluation of CMS and/or CMS risk factor prevalence; and (4) measurement of TL.

### Data extraction

Information gleaned from each study included (1) the study’s aim or purpose; (2) study design; (3) participant characteristics (e.g., mean age, race or ethnicity); (4) CMS risk factors (any or all risk factors: increased WC, elevated BP, impaired FBG, increased TG, and decreased HDL); (5) CMS prevalence; (6) characteristics of telomere analysis, including sample tested and the testing method employed; (7) statistical tests to examine relationship between CMS and TL; (8) study findings regarding TL variations; (9) and subsequent findings identified in follow-up. Two reviewers independently performed data extraction (JP, CS), and any disparities were discussed and resolved through dialogue, ultimately achieving a unanimous consensus.

### Quality assessment

The quality of the included studies was assessed using the Cochrane ROBINS-I (Sterne et al., 2016) tool for non-randomized trials. Though RoB 2 (Sterne et al., 2019) was planned for randomized trials, only observational studies remained after searches were conducted and studies were screened and reviewed for inclusion. Two reviewers (JP, CS) independently conducted a quality assessment, with any discrepancies resolved through discussion. Despite designating a third reviewer (EL) to address unresolved discrepancies, a consensus was reached without necessitating the supplementary reviewer’s intervention in making the final decision.

### Data synthesis and analysis

The Cochrane Data Synthesis and Analysis Narrative Synthesis Guidelines (Ryan & Cochrane Consumers and Communication Review Group, 2013) were followed to synthesize the characteristics and findings of the studies. The synthesis included (1) participant race or ethnicity; (2) participant mean age; (3) CMS prevalence; (4) CMS risk factors (any or all risk factors); (5) characteristics of the telomeres (sample type, analysis method); (6) description of findings for the TL outcome; (7) follow-up findings; (8) strengths; and (9) limitations. The statistical significance of the associations between CMS risk factors and TL was recorded as reported by each study. Effect size estimates were not consistently reported and therefore were not extracted or synthesized. The analytical process involved the following steps: (1) conduct a preliminary narrative synthesis of the findings from the included studies and (2) explore the patterns and relationships within and between data from the studies. Given the heterogeneity of designs and outcomes and the narrative nature of the synthesis, no formal assessment of publication bias was conducted.

A narrative synthesis was performed to investigate the association between CMS and/or CMS risk factors and TL among different age groups of women, as a meta-analysis was deemed inappropriate due to substantial heterogeneity in study designs, populations, measurement methods, and definitions of CMS and TL. Given that the studies did not conduct subgroup analyses based on age, the age groups were determined based on the mean age of the samples. These groups were then classified into distinct life stages informed by life course and menopausal epidemiology: early adulthood (18–45.9 years), corresponding to reproductive years; middle adulthood (46-65 years), which encompasses the perimenopausal and early postmenopausal transition associated with metabolic changes; and later adulthood (>65 years), representing older postmenopausal women at higher risk for cardiometabolic and aging-related conditions (Cankurtaran et al., 2006; Davis et al., 2023; Greendale et al., 2019; Kuokkanen & Santoro, 2001; Moreira et al., 2022). A narrative synthesis was also conducted aiming to investigate variations in CMS risk factors and TL by race or ethnicity. However, due to the absence of subanalyses in the collected studies, studies were grouped by continent as a proxy for potential population and contextual differences, recognizing that this approach may conflate within-continent heterogeneity in race/ethnicity, environment, and healthcare systems. These results should thus be interpreted cautiously and highlight the need for future studies to report and analyze associations stratified by race or ethnicity using standardized categories.

## Results

**Figure 1.**
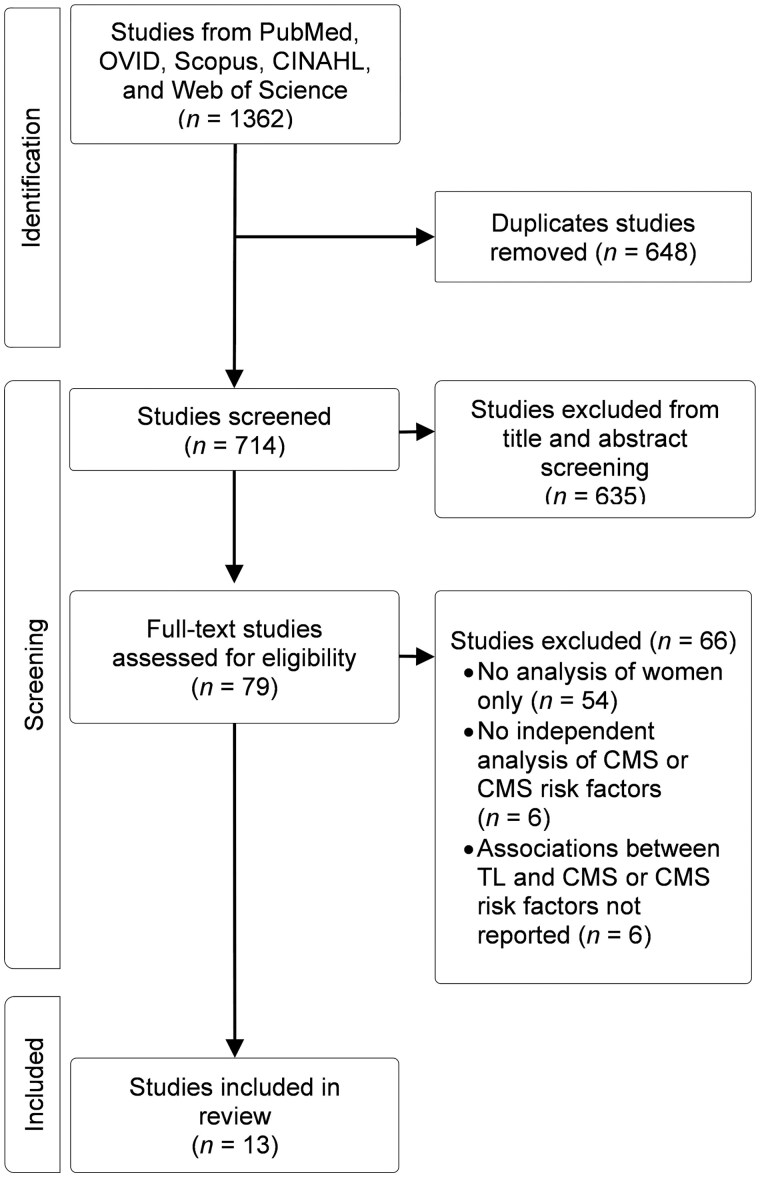
PRISMA flow diagram (Page et al., 2021). CMS = ­cardiometabolic syndrome; TL = telomere length.

The PRISMA flow diagram in Figure 1 outlines the screening and selection procedure for this review. A total of 1,362 initial references were identified in the electronic searches. After screening titles and abstracts, 79 articles underwent a comprehensive full-text review, and 13 articles met the inclusion and exclusion criteria.

### Study characteristics

Study characteristics and findings are summarized in Table 1. Studies were published between 2007 (Bekaert et al., 2007) and 2022 (Bhatt et al., 2022; Ngwa et al., 2022). Ten studies were cross-sectional study designs (Al-Attas et al., 2010; Bekaert et al., 2007; Bhatt et al., 2022; Cheng et al., 2017; Iglesias Molli et al., 2017; Khalangot et al., 2017; Maeda et al., 2011; Mazidi et al., 2018; Ngwa et al., 2022; Nordfjäll et al., 2008), and three studies were cohort designs (Cui et al., 2013; Guzzardi et al., 2015; Kim et al., 2009). Of the cohort study designs, only one study conducted longitudinal analyses (Guzzardi et al., 2015). The remaining two (Cui et al., 2013; Kim et al., 2009) were based on baseline data only and were therefore classified as cross-sectional for the purposes of this review. The significance level was set at 0.05 for all studies.

**Table 1.** Characteristics of included studies.

Authors (year)	Study design	Study setting	Sample		CMS risk factor	TL cell type/ measurement	CMS risk factor/TL significance
			*n* (Mean age)	Race or ethnicity			
**Al-Attas et al. (2010)**	Cross-sectional	Saudi Arabia	96 (39.2)	Arab	WC, BP, FBG, TG, HDL	Leukocytes qPCR	No significant associations between WC, BP, FBG, TG, HDL, and TL
**Bekaert et al. (2007)**	Cross-sectional	Belgium	1,291 (45.9)	Non-Hispanic White	WC, BP, FBG, TG, HDL	Leukocytes Southern blot method	No significant associations between WC, BP, FBG, TG, HDL, and TL
**Bhatt et al. (2022)**	Cross-sectional	North India	797 women (42.0)	Asian Indian	WC, BP, FBG	Leukocytes qPCR	(+) Significant association between increased WC and decreased TL (+) Significant association between increased FBG and decreased TL No significant associations between BP and TL
**Cheng et al. (2017)**	Cross-sectional	United States	3,806 women (50.6)	Mexican American (24.2%); Other Hispanic (5.6%); Non-Hispanic White (50.5%); Non-Hispanic Black (16.4%); Other (3.2%)	WC, BP, FBG, TG, HDL Total number of CMS risk factors	Leukocytes qPCR	(+) Significant association between increased WC and decreased TL (+) Significant association between increased TG and decreased TL (+) Significant association between increased number of CMS risk factors and decreased TL No significant associations between BP, FBG, HDL, and TL
**Cui et al. (2013)**	Cross-sectional	China	2,912 (55.0)	Chinese	WC	Leukocytes qPCR	(+) Significant association between increased WC and decreased TL
**Guzzardi et al. (2015)**	Cohort	Finland	610 (71.1)	NR	WC, BP, FBG, TG, HDL	Leukocytes qPCR	No significant associations between WC, BP, FBG, TG, HDL, and TL
**Iglesias Molli et al. (2017)**	Cross-sectional	South America	400 (46.8)	NR	Total number of CMS risk factors	Leukocytes qPCR	(+) Significant association between the increased number of CMF risk factors and decreased TL
**Khalangot et al. (2017)**	Cross-sectional	Ukraine	62 (62.0)	Non-Hispanic White	WC, BP, FBG	Leukocyte qPCR	(+) Significant association between increased FBG and decreased TL No significant associations between WC, BP, and TL
**Kim et al. (2009)**	Cross-sectional	United States and Puerto Rico	647 (54.5)	Non-Hispanic White (83.2%); Non-White (16.8%)	WC	Leukocytes qPCR	(+) Significant association between increased WC and decreased TL
**Maeda et al. (2011)**	Cross-sectional	Japan	35 (69.7)	Japanese	FBG, TG, HDL	Leukocytes Southern blot	(+) Significant association between increased HDL and increased TL No significant associations between FBG, TG, and TL
**Mazidi et al. (2018)**	Cross-sectional	United States	4,769 (42.6)	Non-Hispanic White (69.9%); Non-Hispanic Black (11.0%); Mexican American (6.6%); Other Hispanic and other (12.4%)	WC, BP, FBG, TG, HDL	Leukocytes qPCR	No significant associations between WC, BP, FBG, TG, HDL, and TL
**Ngwa et al. (2022)**	Cross-sectional	South Africa	445 (43.0)	Black African	WC, BP, FBG, TG, HDL	Leukocytes qPCR	No significant associations between WC, BP, FBG, TG, HDL, and TL
**Nordfjäll et al. (2008)**	Cross-sectional	Sweden	475 (52.4)	NR	WC, BP, FBG, TG, HDL	Leukocytes qPCR	(+) Significant association between increased WC and decreased TL No significant associations between BP, FBG, TG, HDL, and TL

*Note*. Leukocytes refer to DNA extracted from white blood cells isolated from whole blood, without distinguishing between cell subtypes. BP = blood pressure; CMS = cardiometabolic syndrome; FBG = fasting blood glucose; HDL = high-density lipoprotein; NR = not reported; qPCR = quantitative polymerase chain reaction; TG = triglycerides; TL = telomere length; WC = waist circumference.

The study settings encompassed 11 countries, including three (23.1%) studies from the United States (Cheng et al., 2017; Kim et al., 2009; Mazidi et al., 2018) and one study from each of the following countries: Argentina (Iglesias Molli et al., 2017), Belgium (Bekaert et al., 2007), China (Cui et al., 2013), Finland (Guzzardi et al., 2015), India (Bhatt et al., 2022), Japan (Maeda et al., 2011), Saudia Arabia (Al-Attas et al., 2010), South Africa (Ngwa et al., 2022), Sweden (Nordfjäll et al., 2008), and Ukraine (Khalangot et al., 2017).

### Participant characteristics

Sample sizes ranged from 35 to 4,769 women, with 16,345 total women included across all studies. Participants’ ages ranged from 28.2 (Al-Attas et al., 2010) to 90.0 years old (Maeda et al., 2011). The mean age was 51.9, and means ranged from 39.2 (Al-Attas et al., 2010) to 71.1 (Guzzardi et al., 2015) across studies. Studies included women in early (38.5%; Al-Attas et al., 2010; Bekaert et al., 2007; Bhatt et al., 2022; Mazidi et al., 2018; Ngwa et al., 2022), middle (46.2%; Cheng et al., 2017; Cui et al., 2013; ­Iglesias Molli et al., 2017; Khalangot et al., 2017; Kim et al., 2009; Nordfjäll et al., 2008), and later adulthood (15.4%; Guzzardi et al., 2015; Maeda et al., 2011).

Race or ethnicity of the women was reported in ten (76.9%) studies (Al-Attas et al., 2010; Bekaert et al., 2007; Bhatt et al., 2022; Cheng et al., 2017; Cui et al., 2013; Khalangot et al., 2017; Kim et al., 2009; Maeda et al., 2011; Mazidi et al., 2018; Ngwa et al., 2022). The race or ethnic characteristics included non-Hispanic White (Bekaert et al., 2007; Cheng et al., 2017; Khalangot et al., 2017; Kim et al., 2009; Maeda et al., 2011; Mazidi et al., 2018), non-Hispanic Black (Cheng et al., 2017; Mazidi et al., 2018), Black African (Ngwa et al., 2022), Mexican American (Cheng et al., 2017; Mazidi et al., 2018), other Hispanic (Cheng et al., 2017; Mazidi et al., 2018), Chinese (Cui et al., 2013), Asian Indian (Bhatt et al., 2022), Arab (Al-Attas et al., 2010), Japanese (Maeda et al., 2011), and non-White, which included non-Hispanic Black, Hispanic Whites, and other (Kim et al., 2009). Three (23.1%) studies did not specify race or ethnicity (Guzzardi et al., 2015; Iglesias Molli et al., 2017; Nordfjäll et al., 2008).

### CMS risk factor measurement

The operational definitions of the CMS risk factors by study are reported in Supplementary Table 1 (see online supplementary material). Seven unique criteria for CMS risk factor cut off points were identified in this review: National Cholesterol Education Program-Adult Treatment Panel III (NCEP ATP III) CMS criteria, International Diabetes Federation (IDF) CMS criteria, the consensus statement for diagnosing metabolic syndrome in Asian Indians, American Diabetes Association (ADA) criteria for abdominal obesity, the World Health Organization (WHO) criteria for abdominal obesity, the European Society of Cardiology/European Society of Hypertension Guidelines for the management of arterial hypertension for the BP criteria, and the South African Medical Association and Lipid and Atherosclerosis Society of Southern Africa Working Group criteria for measuring TG and HDL.

CMS measurement varied: eight (61.5%; Al-Attas et al., 2010; Bekaert et al., 2007; Cheng et al., 2017; Guzzardi et al., 2015; Iglesias Molli et al., 2017; Mazidi et al., 2018; Ngwa et al., 2022; Nordfjäll et al., 2008) included all five risk factors, three (23.1%; Bhatt et al., 2022; Khalangot et al., 2017; Maeda et al., 2011) included three risk factors, and two (15.4%; Cui et al., 2013; Kim et al., 2009) focused on one. Two studies examined the association between the number of CMS risk factors and TL (15.4%; Cheng et al., 2017; Iglesias Molli et al., 2017); however, only one of those conducted a separate analysis to determine the association between each individual risk factor and TL (Cheng et al., 2017).

### TL measurement

TL was measured using quantitative polymerase chain reaction (qPCR) in most studies (84.6%; 11/13; Al-Attas et al., 2010; Bhatt et al., 2022; Cheng et al., 2017; Cui et al., 2013; Guzzardi et al., 2015; Iglesias Molli et al., 2017; Khalangot et al., 2017; Kim et al., 2009; Mazidi et al., 2018; Ngwa et al., 2022; Nordfjäll et al., 2008), with the majority reporting its validity and reliability (90.9%; 10/11; Bhatt et al., 2022; Cheng et al., 2017; Cui et al., 2013; Guzzardi et al., 2015; Iglesias Molli et al., 2017; Khalangot et al., 2017; Kim et al., 2009; Mazidi et al., 2018; Ngwa et al., 2022; Nordfjäll et al., 2008). Two (15.4%; 2/13; Bekaert et al., 2007; Maeda et al., 2011) used the Southern blot method, which, though it has a higher cost and is more time consuming to process, has higher accuracy, making it the gold standard for TL measurement (Lai et al., 2018).

TL was investigated as a continuous variable in 9 (69.2%) of the 13 studies (Al-Attas et al., 2010; Bekaert et al., 2007; Cheng et al., 2017; Cui et al., 2013; Guzzardi et al., 2015; Iglesias Molli et al., 2017; Kim et al., 2009; Mazidi et al., 2018; Nordfjäll et al., 2008). Three (23.1%) studies reported TL in quartiles (Khalangot et al., 2017; Maeda et al., 2011; Ngwa et al., 2022), and one (7.7%) reported TL in quintiles (Bhatt et al., 2022). Eight (61.5%) studies reported TL in relative length as a relative telomere to single copy gene (T/S) ratio (Bhatt et al., 2022; Cheng et al., 2017; Cui et al., 2013; Guzzardi et al., 2015; Khalangot et al., 2017; Kim et al., 2009; Mazidi et al., 2018; Nordfjäll et al., 2008). Three (23.1%) studies reported TL as an absolute length in kilobase pairs (Al-Attas et al., 2010; Iglesias Molli et al., 2017; Ngwa et al., 2022). Two (15.4%) studies reported the mean TL in terminal restriction fragments (Bekaert et al., 2007; Maeda et al., 2011).

Additionally, the methods for obtaining DNA for TL measurement varied across studies. In all cases, DNA was extracted from leukocytes separated from whole blood, but the degree of cell-type isolation was not consistently specified. Some studies isolated specific leukocyte subsets, while others used the entire leukocyte population; however, this distinction was not differentiated in our analysis and is summarized as leukocytes in Table 1.

### Covariates

A total of 39 covariates were statistically controlled for, ranging from 1 (Maeda et al., 2011) to 14 (Cheng et al., 2017) covariates per study (*M *= 5.08 ± 4.21). The most common covariates included age (*n *= 12; Al-Attas et al., 2010; Bekaert et al., 2007; Bhatt et al., 2022; Cheng et al., 2017; Guzzardi et al., 2015; Iglesias Molli et al., 2017; Khalangot et al., 2017; Kim et al., 2009; Maeda et al., 2011; Mazidi et al., 2018; Ngwa et al., 2022; Nordfjäll et al., 2008) and smoking (*n *= 4; Cheng et al., 2017; Cui et al., 2013; Iglesias Molli et al., 2017; Kim et al., 2009). A breakdown of covariates for each study is detailed in Supplementary Table 2 (see online supplementary material).

### Quality assessment

Most studies (84.6%; 11/13) exhibited some level of moderate risk of bias (Al-Attas et al., 2010; Bekaert et al., 2007; Bhatt et al., 2022; Cheng et al., 2017; Cui et al., 2013; Guzzardi et al., 2015; Iglesias Molli et al., 2017; Khalangot et al., 2017; Kim et al., 2009; Ngwa et al., 2022; Nordfjäll et al., 2008). One (7.1%) study had low risk of bias (Maeda et al., 2011), and one (7.1%) had serious risk of bias (Mazidi et al., 2018). See Supplementary Figure 1 (see online supplementary material) for the Cochrane ROBINS-I traffic light plot and summary plot.

### CMS risk factors and TL findings

Seven of the 13 studies reported significant associations with one or more CMS risk factors and TL in the expected risk direction, with increased waist circumference, blood pressure, triglycerides, or fasting blood glucose associated with shorter TL (53.8%; Bhatt et al., 2022; Cheng et al., 2017; Cui et al., 2013; Iglesias Molli et al., 2017; Khalangot et al., 2017; Kim et al., 2009; Nordfjäll et al., 2008), while higher HDL was associated with longer TL in one study (7.7%; Maeda et al., 2011). Five of the studies did not report any significant associations between CMS risk factors and TL (38.5%; Al-Attas et al., 2010; Bekaert et al., 2007; Guzzardi et al., 2015; Mazidi et al., 2018; Ngwa et al., 2022).

Increased waist circumference (WC) was the most consistently associated CMS component, with five studies (38.5%; Bhatt et al., 2022; Cheng et al., 2017; Cui et al., 2013; Kim et al., 2009; Nordfjäll et al., 2008) reporting significant inverse associations between increased WC and shorter TL. FBG was inversely associated in two studies (15.4%; Bhatt et al., 2022; Khalangot et al., 2017), elevated TG was associated with shorter TL in one study (7.7%; Cheng et al., 2017), and higher HDL was associated with longer TL in one (7.7%; Maeda et al., 2011). BP showed no significant associations in any study. Finally, both studies examining the total number of CMS risk factors reported significant inverse associations with TL (15.4%; Cheng et al., 2017; Iglesias Molli et al., 2017). Full study-level results, including non-significant findings, are presented in Table 2.

**Table 2.** Significant findings by CMS risk factors.

CMS/risk factor	Total *n*	Significant *n*
**Waist circumference**	11	5
**Blood pressure**	9	0
**Fasting blood glucose**	10	2
**Triglycerides**	8	1
**High-density lipoproteins**	8	1
**Total number of risk factors**	2	2

*Note*. CMS = cardiometabolic syndrome.

### CMS risk factors and/or CMS and TL by mean age

Overall significant findings by mean age group can be found in Table 3, and significant findings by mean age group and CMS risk factors in Table 4. Among studies classified as early adulthood (*n* = 5), only one (20%; Bhatt et al., 2022) reported a significant association between CMS risk factors and TL (Al-Attas et al., 2010; Bekaert et al., 2007; Mazidi et al., 2018; Ngwa et al., 2022). In later adulthood (*n* = 2), one study (50%; Maeda et al., 2011) reported significant findings (Guzzardi et al., 2015). In contrast, all six studies of middle adulthood reported significant associations (Cheng et al., 2017; Cui et al., 2013; Iglesias Molli et al., 2017; Khalangot et al., 2017; Kim et al., 2009; Nordfjäll et al., 2008).

**Table 3.** Significant findings by mean age.

Life stage	Total *n*	Significant *n*
**Early adulthood**	5	1
**Middle adulthood**	6	6
**Later adulthood**	2	1

*Note*. Early adulthood: 18–45.9 years; middle adulthood: 46–65 years; later adulthood: >65 years.

**Table 4.** Significant findings by mean age and CMS risk factor.

Life stage	WC		BP		FBG		TG		HDL		Total CMS risk factors	
	Total	Significant	Total	Significant	Total	Significant	Total	Significant	Total	Significant	Total	Significant
	*n*	*n*	*n*	*n*	*n*	*n*	*n*	*n*	*n*	*n*	*n*	*n*
**Early adulthood**	5	1	5	0	5	1	4	0	4	0	-	-
**Middle adulthood**	5	4	3	0	3	1	2	1	2	0	2	2
**Later adulthood**	1	0	1	0	2	0	2	0	2	1	-	-
**Total**	11	5	9	0	9	2	8	1	8	1	2	2

*Note*. BP = blood pressure; CMS = cardiometabolic syndrome; FBG = fasting blood glucose; HDL = high-density lipoproteins; TG = triglycerides; WC = waist circumference; early adulthood: 18–45.9 years; middle adulthood: 46–65 years; later adulthood: >65 years; (-) = no applicable studies for this category; (0) = no significant findings demonstrated.

For WC specifically, one early adulthood study (20%; Bhatt et al., 2022) and four middle adulthood studies (80%; Cheng et al., 2017; Cui et al., 2013; Kim et al., 2009; Nordfjäll et al., 2008) reported significant inverse associations, whereas neither later adulthood study did (Guzzardi et al., 2015). No significant associations between BP and TL were reported in any age group (Al-Attas et al., 2010; Bekaert et al., 2007; Bhatt et al., 2022; Cheng et al., 2017; Guzzardi et al., 2015; Khalangot et al., 2017; Mazidi et al., 2018; Ngwa et al., 2022; Nordfjäll et al., 2008).

For FBG, significant inverse associations were observed in one early adulthood study (20%; Bhatt et al., 2022) and one middle adulthood study (33%; Khalangot et al., 2017) (Al-Attas et al., 2010; Bekaert et al., 2007; Cheng et al., 2017; Maeda et al., 2011; Mazidi et al., 2018; Ngwa et al., 2022; Nordfjäll et al., 2008). For TG, only one middle adulthood study (50%; Cheng et al., 2017) reported a significant association (Nordfjäll et al., 2008), and for HDL, one later adulthood study (50%; Maeda et al., 2011) reported a significant positive association (Guzzardi et al., 2015), with no significant associations in earlier age groups (Al-Attas et al., 2010; Bekaert et al., 2007; Cheng et al., 2017; Mazidi et al., 2018; Ngwa et al., 2022; Nordfjäll et al., 2008). See Table 5 for the summary of findings by life stage.

**Table 5.** Summary of findings by life stage.

Age group	WC	BP	FBG	TG	HDL	CMS (all factors)
**Early adulthood (18-45.9)**	↑ WC → ↓ TL (Bhatt et al., 2022; Cheng et al., 2017)	None	↑ FBG → ↓ TL (Bhatt et al., 2022)	None	None	None
**Middle adulthood (46-65)**	↑ WC → ↓ TL (Cheng et al., 2017; Cui et al., 2013; Kim et al., 2009; Nordfjäll et al., 2008)	None	↑ FBG → ↓ TL (Khalangot et al., 2017)	↑ TG → ↓ TL (Cheng et al., 2017)	None	↑ CMS risk factors → ↓ TL (Cheng et al., 2017; Iglesias Molli et al., 2017)
**Later adulthood (>65)**	None	None	None	None	↑ HDL → ↑ TL (Maeda et al., 2011)	None

*Note*. BP = blood pressure; CMS = cardiometabolic syndrome; FBG = fasting blood glucose; HDL = high-density lipoprotein; TG = triglycerides; TL = telomere length; WC = waist circumference.

### CMS risk factors and/or CMS and TL by race or ethnicity

An examination of CMS risk factors and/or CMS TL findings by race or ethnicity was not conducted in any of the included studies. Three (30%) of the ten studies that included race or ethnicity in the demographics had heterogeneous, multiracial, or multiethnic populations and statistically adjusted or controlled for the feature (Cheng et al., 2017; Kim et al., 2009; Mazidi et al., 2018). The remaining seven (70%; 7/10) studies featured homogenous populations from a single area (Al-Attas et al., 2010; Bekaert et al., 2007; Bhatt et al., 2022; Cui et al., 2013; Khalangot et al., 2017; Maeda et al., 2011; Ngwa et al., 2022).

Supplementary Table 3 (see online supplementary material) summarizes findings by continent, and Supplementary Table 4 (see online supplementary material) provides significant findings by CMS risk factors and continent. Significant findings were in 75% (3/4) of the studies from Asia, 50% (2/4) of European countries, 100% (1/1) of South American studies, and 66.7% (2/3) of studies from North America. For WC, significant inverse associations with TL were observed in 66.7 % of studies from Asia (Bhatt et al., 2022; Cui et al., 2013; cf Al-Attas et al., 2010), 25 % from Europe (Nordfjäll et al., 2008; cf Bekaert et al., 2007; Guzzardi et al., 2015; Khalangot et al., 2017), and 66.7 % from North America (Cheng et al., 2017; Kim et al., 2009; cf Mazidi et al., 2018), while no significant association was reported in Africa (Ngwa et al., 2022). No studies from any continent reported significant associations between BP and TL (Al-Attas et al., 2010; Bekaert et al., 2007; Bhatt et al., 2022; Cheng et al., 2017; Guzzardi et al., 2015; Khalangot et al., 2017; Mazidi et al., 2018; Ngwa et al., 2022; Nordfjäll et al., 2008).

For FBG, significant inverse associations were reported in 33.3 % of Asian studies (Bhatt et al., 2022; cf Al-Attas et al., 2010; Maeda et al., 2011) and 25 % of European studies (Khalangot et al., 2017; cf Bekaert et al., 2007; Guzzardi et al., 2015; Nordfjäll et al., 2008), but none in Africa (Ngwa et al., 2022) or North America (Cheng et al., 2017; Mazidi et al., 2018). Elevated TG was significantly associated with shorter TL in 50 % of North American studies (Cheng et al., 2017; cf Mazidi et al., 2018), with no significant findings in Africa, Asia, or Europe. For HDL, a significant positive association with TL was observed in 50 % of Asian studies (Maeda et al., 2011; cf Al-Attas et al., 2010), but none in Africa, Europe, or North America. Finally, total CMS risk factors were significantly associated with shorter TL in the only studies from South America (Iglesias Molli et al., 2017) and North America (Cheng et al., 2017) that examined this association.

## Discussion and implications

This systematic review investigated the association between CMS and CMS risk factors and TL among women, addressing an important gap in the literature. Unlike previous reviews that focused on individual CMS components or mixed-sex populations, this study uniquely examined the collective impact of all five CMS risk factors as well as CMS as a clinical syndrome, specifically in women. Age and race or ethnicity were also considered. Although associations between individual CMS risk factors and TL were inconclusive, some evidence suggested a relationship between CMS as a syndrome and TL shortening. When disaggregating findings by mean age, increased WC appeared to be related to decreased TL in middle adulthood. Findings by race or ethnicity were inconclusive due to a lack of subanalyses; however, when findings were grouped by study continent, a relationship between increased WC and decreased TL was found in studies conducted in Asia and North America.

The overall findings of this review did not provide evidence of an association between individual CMS risk factors and TL in women, which was unexpected. These findings conflict with prior literature that reported a significant association between increased WC (Lee et al., 2011) and increased FBG (Demissie et al., 2006) and shorter TL. The contrasting findings may stem from variability in how CMS risk factors were operationalized and differences in study designs, populations, and adjustment for medication use. For example, eight of the eleven studies that examined BP reported controlling for use of anti-hypertensive meds, while six of the eleven studies examining FBG controlled for use of antidiabetic medications, potentially underestimating associations. Studies applied a range of diagnostic criteria for CMS risk factors, including ADA, IDF, NCEP ATP III, and WHO definitions, further contributing to heterogeneity and limiting the generalizability of findings. Such differences reflect variations in study design, population, and research objectives, complicating direct comparisons and limiting the strength of conclusions (Naaktgeboren et al., 2016).

Furthermore, variations in race or ethnicity among the study populations may also account for these inconsistent findings. Some of the studies that did not find significant associations included more racially or ethnically diverse populations (Al-Attas et al., 2010; Bhatt et al., 2022; Cui et al., 2013; Maeda et al., 2011; Ngwa et al., 2022), which might reflect differing genetic or environmental influences on TL. These inconsistencies underscore the challenge of determining associations between CMS risk factors and TL in women, stressing the need for consensus on operationalizing and measuring CMS risk factors.

Evidence also suggested an association between CMS and TL shortening with the collective consideration of all CMS risk factors, though this finding was limited, with only two studies. The observed relationship in this review could be attributed to an accelerated TL attrition due to the increased oxidative stress from the cumulative effect of all CMS risk factors when assessed collectively (von Zglinicki, 2002), suggesting that TL is not just a transient marker of metabolic health decline, but rather an indicator of long-term, chronic deterioration in metabolic health (Révész et al., 2014). This was supported in prior literature in participants with shorter TL from baseline measures who tended to experience more pronounced progression in the number of CMS components over time (Révész et al., 2014). However, the few studies that have explored the relationship between the number of CMS risk factors and TL have not delved into this association’s longitudinal dynamics (Cheng et al., 2017; Iglesias Molli et al., 2017).

Increased WC was related to decreased TL for women in middle adulthood but not for women in early or later adulthood. While it has been well established that TL decreases with age (Shammas, 2011), the lack of significant findings for WC in early adulthood, compared to the significant associations observed in middle adulthood, is particularly noteworthy. This difference may be due to hormonal fluctuations associated with the perimenopausal changes that occur during middle adulthood (Motlani et al., 2023; Pataky et al., 2021). Notably, the loss of estrogen, which begins to fluctuate unpredictably during perimenopause before falling to very low levels in menopause (Randolph et al., 2004; Yu et al., 2022), has been linked to weight gain and increased WC (Chopra et al., 2019). Estrogen also plays a role in protecting TL (Kresovich et al., 2018), with prior research indicating that it may help preserve telomeres through its antioxidant effects (Viña et al., 2005).

Investigation into the relationship between CMS and TL by race or ethnicity remains limited. Grouping findings by continent suggested potential population-level differences with increased WC and decreased TL in studies from Asia and North America, but these findings should be interpreted with caution because continent-level analyses conflate genetic, environmental, and social heterogeneity (Mensah et al., 2023; Noubiap et al., 2022). It has also been established that physiological and genetic differences in body composition and metabolic function specific to sex and race or ethnicity may also influence TL (Bekaert et al., 2007; Haycock et al., 2014). With WC specifically, prior research has highlighted substantial variations in WC and other obesity-related measures among racial and ethnic groups (Deurenberg et al., 1998; Flegal et al., 2009, 2010; Misra & Khurana, 2009). This review echoed these differences, with several studies indicating the need for adjustments in CMS criteria to account for the variations, which may otherwise lead to misdiagnosis of CMS (Bhatt et al., 2022; Cui et al., 2013; Misra & Khurana, 2009; Misra et al., 2019). Despite efforts to adjust CMS criteria for racial and ethnic specificities (Alberti et al., 2009; WHO Expert Consultation, 2004), a consensus remains elusive, limiting the findings’ applicability.

Future research should focus on large, longitudinal cohort studies that address these gaps by using standardized definitions of CMS, including measures of hormonal and inflammatory pathways, and exploring how associations differ by age, race, and ethnicity. These studies could improve our understanding of causal relationships, highlight when in life interventions might be most effective, and help shape prevention efforts to lower cardiometabolic risk and support healthy aging in women.

### Implications

Researchers are called upon to establish a global consensus on the operationalization of CMS diagnostic criteria and to design longitudinal studies to clarify temporal and causal relationships between CMS risk factors and telomere length. Large cohort studies are needed to determine age- and race- or ethnicity-specific differences in CMS risk factor measures, and to identify critical windows of susceptibility, such as middle adulthood, when interventions may be most impactful. Clinicians and public health practitioners can use waist circumference as a simple, low-cost risk stratification tool to identify women at higher risk of accelerated aging and cardiometabolic disease. Programs designed to reduce abdominal fat by promoting healthy eating patterns, regular exercise, and supportive behavioral strategies could help mitigate cardiometabolic risk and preserve healthy aging in middle-aged women. Funders are encouraged to support global research initiatives that explore variability in CMS risk factors and their measurement across diverse populations of women. Policymakers should champion the development of standardized diagnostic criteria for CMS that are sensitive to sex, age, and race or ethnicity, while also supporting the integration of waist circumference screening and tailored interventions into preventive care guidelines for women.

## Strengths and limitations

This review is the first to examine the association between CMS or CMS risk factors and TL in women. The methodology utilized in this review implemented a broad, inclusive approach, not setting restrictions by publication date, study design, participant age and/or race or ethnicity, study continent, TL measurement, or analytic methods. This approach allowed for a more comprehensive review, which enhanced diversity in the findings.

However, the predominance of cross-sectional studies limits causal inference, emphasizing the need for longitudinal randomized controlled trials and cohort studies to better understand the relationship between CMS risk factors and TL. None of the included studies examined potential mediating or moderating pathways, such as the influence of central adiposity, hormonal changes during midlife, or inflammatory processes, which may help explain the observed associations between CMS risk factors and TL. The lack of subanalyses by race and ethnicity across studies is also notable, as these factors may modify the observed associations and are necessary components for developing equitable, population-specific prevention strategies. In addition, heterogeneity in how CMS risk factors were defined and measured limits comparison and generalizability of the findings. Furthermore, publication bias could not be formally assessed due to the heterogeneity of study designs, the absence of consistent effect size estimates, and the narrative synthesis approach. Future studies should incorporate analytic approaches to test these mechanisms, which could clarify how CMS risk factors contribute to telomere attrition in women. Lastly, a formal meta-analysis was not conducted due to significant heterogeneity among the included studies in design, population characteristics, measurement techniques for TL, and the definition and assessment of CMS and its risk factors, which limited our ability to draw overall conclusions typical of a standard meta-analysis (Greco et al., 2013; Imrey, 2020). Consequently, our findings are based on narrative synthesis, which, while insightful, may not provide the quantitative precision that a meta-analysis could offer (Lisy & Porritt, 2016).

## Conclusions

Overall, this review did not find a relationship between individual CMS risk factors and TL in women. The evidence did suggest a negative association between the total number of CMS risk factors present and TL in women and WC and TL in women in middle adulthood. Future research should prioritize large, longitudinal cohort studies that explore the associations between CMS risk factors and TL in women throughout the lifespan, focusing on determining age-specific and racial or ethnic variations, as well as identifying standardized criteria to define CMS in women. Building this evidence will help shape more precise and effective prevention strategies for women.

## Supplementary Material

igaf091_Supplementary_Data

## Data Availability

This systematic review is registered with the International Prospective Register of Systematic Reviews (CRD42023407309).
