# Supplementary material for: Examining the relationship between cardiometabolic risk factors and telomere length in women: a systematic review
Source: Innov Aging. 2025 Aug 25;9(9):igaf091. doi: 10.1093/geroni/igaf091 (PMC12505143; doi:10.1093/geroni/igaf091)

***Innovation in Aging* Supplementary Material:** **Page, Stephens, Richard, Lyons, Baumler, Verklan, & Lorenzo. Examining the relationship between cardiometabolic risk factors and telomere length in women: A systematic review.**

| **Supplementary Table 1.** *Operationalization of CMS Risk Factor by Study* | | | | | | |
| --- | --- | --- | --- | --- | --- | --- |
| **Authors (year)** | **CMS risk factor criteria** | **CMS risk factor measurement/comparison** | | | | |
|  |  | **WC** | **BP** | **FBG** | **TG** | **HDL** |
| Al-Attas et al. (2010) | NA | Continuous (cm) | Continuous (mm Hg) | Continuous (mmol/l) | Continuous (mmol/l) | Continuous (mmol/l) |
| Bekaert et al. (2007) | NA | Continuous (cm) | Continuous (mm Hg);  Dichotomous (mm Hg):  SBP < 140 vs ≥ 140;  DBP < 90 vs ≥ 90 or treated for HTN | Continuous (mg dL^-1^) | Continuous (mg dL^-1^) | Continuous (mg dL^-1^) |
| Bhatt et al. (2022) | Consensus statement for diagnosing MetSyn in Asian Indians^a^ | Continuous (cm) | Dichotomous (mm Hg):  SBP < 130 vs ≥ 130; DBP < 85 vs ≥ 85 | Dichotomous (mg/dL):  ≤ 100 vs > 100 | NA | NA |
| Cheng et al. (2017) | NCEP ATP III | Dichotomous (cm): normal vs ≥ 90^th^ percentile for age and sex | Dichotomous (mm Hg): normal SBP or DBP vs ≥ 90^th^ percentile for age, sex, height, or treated for HTN | Dichotomous (mm/dL):  < 100 vs ≥ 100 or treated for DM | Dichotomous (mg/dL): < 110 vs ≥ 110 | Dichotomous (mg/dL)  ≤ 40 vs > 40 |
| Cui et al. (2013) | ADA | Categorical (cm): Normal (<80) vs action level 1 (80-87.9) vs action level 2 (≥ 88) | NA | NA | NA | NA |
| Guzzardi et al. (2015) | NA | Continuous (cm) | Continuous (mm Hg) | Continuous (mM) | Continuous (mmol/L) | Continuous (mmol/L) |
| Iglesias Molli et al. (2017) | NCEP ATP III | Dichotomous (cm):  < 88 vs ≥ 88 | Dichotomous (mm Hg): SBP < 130 vs ≥ 130;  DBP < 85 vs ≥ 85 or treated for HTN | Dichotomous (mm dl^-1^):  < 100 vs ≥ 100 or treated for DM | Dichotomous (mg dl^-1^):  < 150 vs ≥ 150 or treated for dyslipidemia | Dichotomous (mg dl^-1^)  < 50 vs ≥ 50 |
| Khalangot et al. (2017) | IDF | Dichotomous (cm):  < 80 vs ≥ 80 | Dichotomous (mm Hg): SBP < 130 vs ≥ 130;  DBP < 85 vs ≥ 85 or treated for HTN | Categorical (mmol/l)  Normal (< 6.1) vs impaired (≥ 6.1 but < 7.0) vs screen-detected DM (≥ 7.0) | NA | NA |
| Kim et al. (2009) | ADA | Categorical (cm): Normal (<80) vs action level 1 (80-87.9) vs action level 2 (≥ 88) | NA | NA | NA | NA |
| Maeda et al. (2011) | NA | NA | NA | Continuous (mg/dL) | Continuous (mg/dL) | Continuous (mg/dL) |
| Mazidi et al. (2018) | NCEP ATP III | Dichotomous (cm): <88 vs ≥ 88 | Dichotomous (mm Hg): SBP < 130 vs ≥ 130;  DBP < 85 vs ≥ 85 | Dichotomous (mm/dl):  < 100 vs ≥ 100 | Dichotomous (mg/dl):  < 150 vs ≥ 150 | Dichotomous (mg/dl):  < 50 vs ≥ 50 |
| Ngwa et al. (2022) | WHO^b,c^, ESC^d^, SAMA/LASSA^e^ | Dichotomous (cm):  ≤ 80 vs > 80 | Dichotomous (mm Hg):  SBP < 140 vs ≥ 140;  DBP < 90 vs ≥ 90 or treated for HTN | Dichotomous (mmol/L):  <7.0 vs ≥ 7.0 or treated for DM | Dichotomous (mmol/L):  ≤ 1.5 vs > 1.5 or treated for dyslipidemia | Dichotomous (mmol/L):  < 1.2 vs ≥ 1.2 |
| Nordfjäll et al. (2008) | NA | Continuous (cm) | Continuous (mm Hg) | Continuous (mmol/l) | Continuous (mmol/l) | Continuous (mmol/l) |
| *Note*. ADA = American Diabetes Association; BP = blood pressure; cm = centimeters; CMS = cardiometabolic syndrome; DBP = diastolic blood pressure; DM = diabetes; ESC = European Society of Cardiology; FBG = fasting blood glucose; HDL = high-density lipoprotein; HTN = hypertension; IDF = International Diabetes Federation; LASSA = Lipid and Atherosclerosis Society of Southern Africa; mM = millimolar; mg dL⁻¹ = milligrams per deciliter; mg/dL = milligrams per deciliter; mm Hg = millimeters of mercury; mmol/l = millimoles per liter; NA = not applicable; NCEP ATP III = National Cholesterol Education Program Adult Treatment Panel III; SAMA = South African Medical Association; SBP = systolic blood pressure; TG = triglycerides; WC = waist circumference; WHO = World Health Organization  ^a^ Misra, A., Chowbey, P., Makkar, B. M., et al. (2009). Consensus statement for diagnosis of obesity, abdominal obesity and the metabolic syndrome for Asian Indians and recommendations for physical activity, medical and surgical management. *The Journal of the Association of Physicians of India*, *57*, 163–170.  ^b^ World Health Organization (1999) Definition, diagnosis and classification of diabetes mellitus and its complications, Part 1: Diagnosis and classification of diabetes mellitus. Report of a WHO consultation. Department of Non communicable Disease Surveillance, WHO, Geneva.  ^c^ World Health Organization (2011) Waist circumference and waist-hip ratio: Report of a WHO Expert Consultation, Geneva, 8-11 December 2008.  ^d^ Williams, B., Mancia, G., Spiering, W., et al. (2018). 2018 ESC/ESH Guidelines for the management of arterial hypertension. *European Heart Journal*, *39*(33), 3021–3104. https://doi.org/10.1093/eurheartj/ehy339  ^e^ Diagnosis, management and prevention of the common dyslipidaemias in South Africa--clinical guideline, 2000. South African Medical Association and Lipid and Atherosclerosis Society of Southern Africa Working Group. (2000). *South African Medical Journal*, *90*(2 Pt 2), 164–178. | | | | | | |

| **Supplementary Table 2.** *Covariates of Included Studies* | | | | | | | | | |
| --- | --- | --- | --- | --- | --- | --- | --- | --- | --- |
| **Authors (year)** | **Total covariates** | **Age** | **Smoking** | **CRP** | **Education** | **PA** | **Race or ethnicity** | **WC** | **Other covariates** |
| Al-Attas et al. (2010) | 14 | ✓ |  | ✓ |  |  |  | ✓ | Adiponectin, aPAI-1, ANG II, BMI, DBP, HC, leptin, resistin, SBP, TNF-α, use of medications (anti-HTN, anti-DM) |
| Bekaert et al. (2007) | 3 | ✓ |  |  |  |  |  |  | Current use of hormonal contraceptives, use of medications (anti-HTN, ASA, NSAIDs, statins) |
| Bhatt et al. (2022) | 5 | ✓ |  |  | ✓ |  |  |  | Family income, HTN, use of medications (anti-HTN) |
| Cheng et al. (2017) | 14 | ✓ | ✓ | ✓ |  |  | ✓ | ✓ | Alb, ALT, BP, comorbidities, FBG, LDL, total bilirubin, TC, uric acid |
| Cui et al. (2013) | 6 |  | ✓ |  | ✓ |  |  |  | Age at blood collection, alcohol use, case/control status, comorbidities |
| Guzzardi et al. (2015) | 2 | ✓ |  |  |  |  |  |  | Use of medications (anti-HTN, lipid lowering) |
| Iglesias Molli et al. (2017) | 3 | ✓ | ✓ |  |  | ✓ |  |  |  |
| Khalangot et al. (2017) | 3 | ✓ |  |  |  |  |  | ✓ | SBP |
| Lee et al. (2005) | 8 | ✓ |  | ✓ |  | ✓ |  |  | FBG, HDL, pulse pressure, TAS, use of medication (vitamins) |
| Maeda et al. (2011) | 1 | ✓ |  |  |  |  |  |  |  |
| Mazidi et al. (2018) | 2 | ✓ |  |  |  |  | ✓ |  |  |
| Ngwa et al. (2022) | 2 | ✓ |  |  |  |  |  |  | BMI |
| Nordfjäll et al. (2008) | 3 | ✓ |  |  |  |  |  |  | Center, weight |
| Total |  | 12 | 3 | 3 | 3 | 3 | 3 | 3 |  |

*Note*. Alb = albumin; ALT = alanine aminotransferase; ANG II = angiotensin II; ASA = acetylsalicylic acid; aPAI-1 = plasminogen activator inhibitor-1; BMI = body mass index; BP = blood pressure; CRP = C-reactive protein; CVD = cardiovascular disease; DBP = diastolic blood pressure; DM = diabetes; FBG = fasting blood glucose; HC = hip circumference; HDL = high-density lipoprotein; HTN = hypertension; LDL = low-density lipoprotein; NSAIDs = nonsteroidal anti-inflammatory drugs; PA = physical activity; SBP = systolic blood pressure; TAS = total antioxidant status; TC = total cholesterol; TNF-α = tumor necrosis factor-alpha; WC = waist circumference.

| **Supplementary Table 3** | | |
| --- | --- | --- |
| **Continent** | **Total**  ***n*** | **Significant**  ***n*** |
| Africa | 1 | 0 |
| Asia | 4 | 3 |
| Europe | 4 | 2 |
| North America | 3 | 2 |
| South America | 1 | 1 |

| **Supplementary Table 4**  *Significant Findings by Continent and CMS Risk Factor* | | | | | | | | | | | | | |
| --- | --- | --- | --- | --- | --- | --- | --- | --- | --- | --- | --- | --- | --- |
| **Continent** | **WC** | | **BP** | | **FBG** | | **TG** | | **HDL** | | **Total CMS risk factors** | |  |
|  | **Total**  ***n*** | **Significant**  ***n*** | **Total**  ***n*** | **Significant**  ***n*** | **Total**  ***n*** | **Significant**  ***n*** | **Total**  ***n*** | **Significant**  ***n*** | **Total**  ***n*** | **Significant**  ***n*** | **Total**  ***n*** | **Significant**  ***n*** |  |
| Africa | 1 | 0 | 1 | 0 | 1 | 0 | 1 | 0 | 1 | 0 | - | - |  |
| Asia | 3 | 2 | 2 | 0 | 3 | 1 | 2 | 0 | 2 | 1 | - | - |  |
| Europe | 4 | 1 | 4 | 0 | 4 | 1 | 3 | 0 | 3 | 0 | - | - |  |
| North America | 3 | 2 | 2 | 0 | 2 | 0 | 2 | 1 | 2 | 0 | 1 | 1 |  |
| South America | - | - | - | - | - | - | - | - | - | - | 1 | 1 |  |
| *Note.* BP = blood pressure; CMS = cardiometabolic syndrome; FBG = fasting blood glucose; HDL = high-density lipoproteins; TG = triglycerides; WC = waist circumference; (-) = no applicable studies for this category; (0) = no significant findings demonstrated. | | | | | | | | | | | | |  |

**Supplementary Figure 1**

*Cochrane ROBINS-I Traffic Light Plot and Summary Plot*


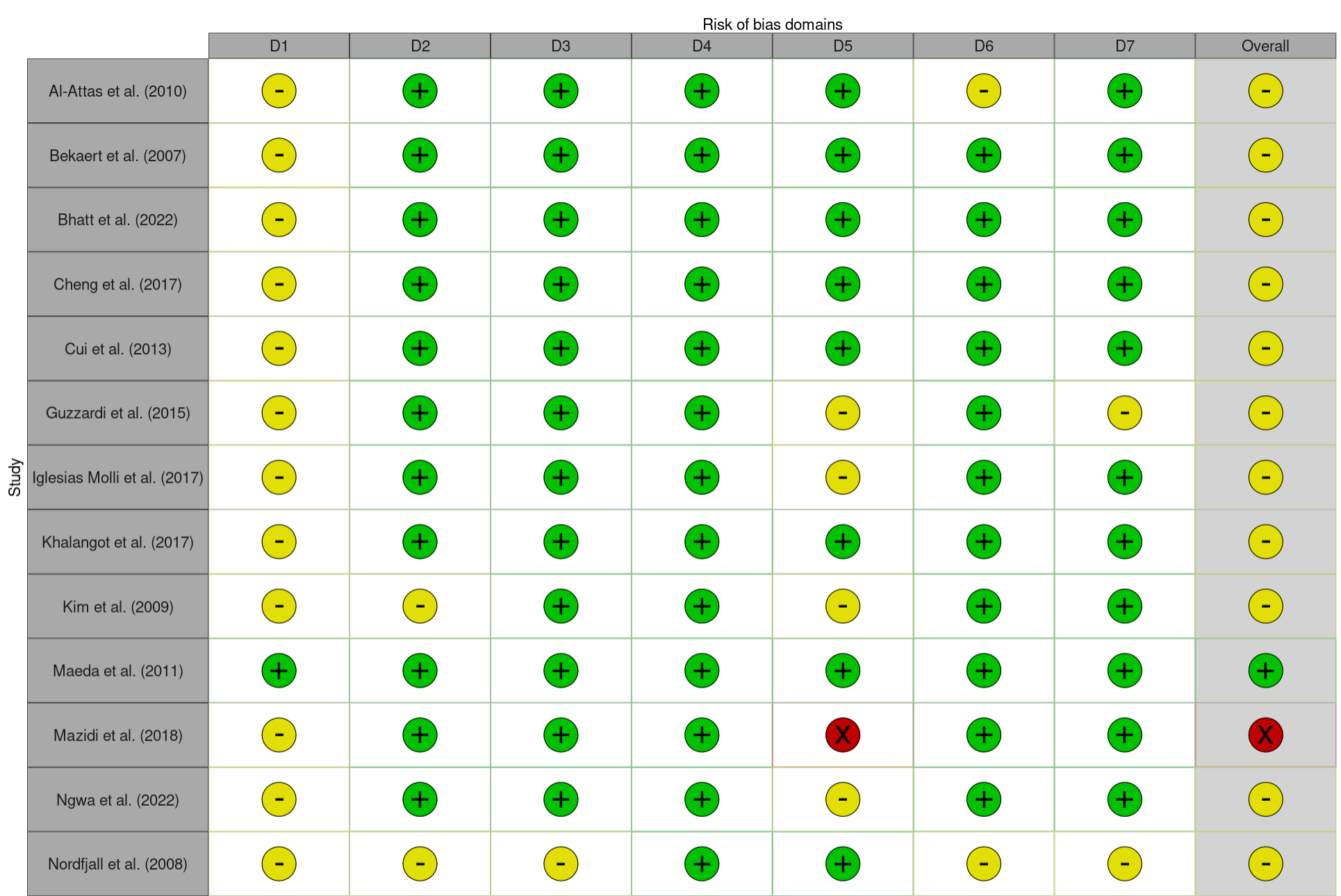


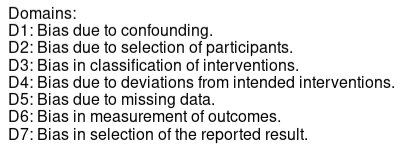

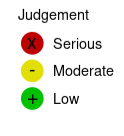

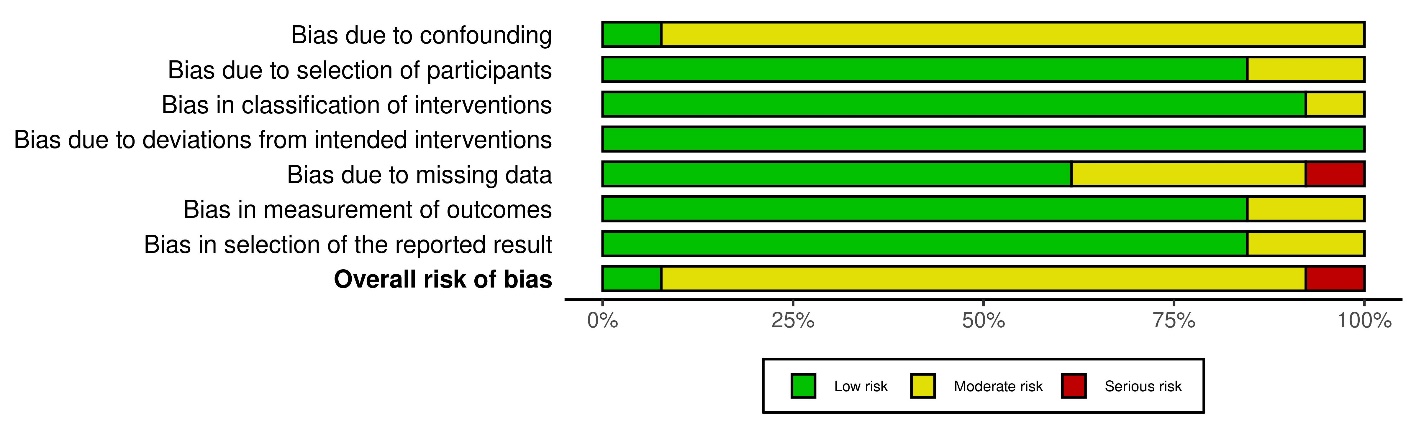

Supplement: igaf091_Supplementary_Data [file igaf091_supplementary_data.docx]
